# Ibrutinib plus fludarabine, cyclophosphamide and rituximab (iFCR) as initial treatment in chronic lymphocytic leukemia/ small lymphocytic leukemia with or without TP53 aberrations: a prospective real-world study in Chinese cohort

**DOI:** 10.1038/s41408-023-00890-y

**Published:** 2023-08-09

**Authors:** Yi Miao, Yeqin Sha, Yi Xia, Shuchao Qin, Rui Jiang, Luomengjia Dai, Hui Shen, Tonglu Qiu, Wei Wu, Jingyan Qiu, Yilian Yang, Chongyang Ding, Yujie Wu, Lei Fan, Wei Xu, Jianyong Li, Huayuan Zhu

**Affiliations:** 1 Department of Hematology, the First Affiliated Hospital of Nanjing Medical University, Jiangsu Province Hospital, 210029 Nanjing, Jiangsu China; 2 Pukou Chronic Lymphocytic Leukemia (CLL) Center, Pukou division of Jiangsu Province Hospital, 211800 Nanjing, Jiangsu China; 3 Jiangsu Key Lab of Cancer Biomarkers, Prevention and Treatment, Collaborative Innovation Center for Cancer Personalized Medicine, Nanjing Medical University, 210029 Nanjing, Jiangsu China; 4 Department of Nuclear Medicine, the First Affiliated Hospital of Nanjing Medical University, Jiangsu Province Hospital, 210029 Nanjing, Jiangsu China

**Keywords:** Chronic lymphocytic leukaemia, Translational research

**Dear Editor,**

The continuous treatment model of Bruton tyrosine kinase inhibitors (BTKi) in chronic lymphocytic leukemia/small lymphocytic leukemia (CLL/SLL) has led to several concerns like lack of deep remission, the potential for inducing resistance mutations, increasing risk of toxic effects and substantial cost [1, 2]. The combination of ibrutinib and chemoimmunotherapy (CIT) was utilized to explore the fix-duration strategies in first-line treatment of CLL patients, achieving a high undetectable minimal residual disease (uMRD) rate and sustaining long-term remission after ibrutinib discontinuation [3–6]. Nevertheless, due to limited cohorts reported, the optimal patients who can potentially achieve early deep remission and benefit from such combinations remained largely unknown. Therefore, we conducted a single-arm, real-world prospective study that analyzed the efficacy and safety of first-line ibrutinib plus fludarabine, cyclophosphamide, and rituximab (iFCR) treatment in CLL/SLL patients with various biological characters including unmutated IGHV status, TP53 aberrations and complex karyotype (CK).

The study enrolled CLL/SLL patients who planned to receive iFCR as first-line therapy at Jiangsu Province Hospital between January 2019 and March 2021. A 0–7-day lead-in period with single-agent ibrutinib (420 mg daily) was feasibly allowed, followed by intravenous rituximab (375 mg/m^2^ cycle 1 and 500 mg/m^2^ cycle 2–6 on day 0), fludarabine (25 mg/m^2^, days 1–3) and cyclophosphamide (250 mg/m^2^, days 1–3), every 28-day cycle, up to a maximum of 6 cycles according to the response and tolerance of patients. Ibrutinib was given continuously after 6 cycles and discontinued in patients who achieved complete remission or complete remission with incomplete count recovery (CR/CRi) and bone marrow (BM) uMRD two years after iFCR initiation. This study was approved by the Institutional Research Board of Jiangsu Province Hospital and was carried out following the Declaration of Helsinki. The primary outcome was the rate of CR/CRi with BM-uMRD. Response assessments were conducted after 3 cycles of iFCR (C4D0), 2 months after 6 cycles of combined therapy (C9D0), and then after 12 cycles (C13D0), 18 cycles (C19D0), 24 cycles (C25D0), and 30 cycles (C31D0). uMRD was defined as less than one CLL cell per 10,000 leukocytes by flow cytometry (FCM). MRD results negative at 10^−6^, 10^−5^, 10^−4^, 10^−3^, 10^−2^, 10^−1^, and ≥10% levels were defined as MRD6, MRD5, MRD4, MRD3, MRD2, MRD1, and MRD0, respectively. MRD recurrence was defined as two consecutive values of ≥0.01% after achieving uMRD [7]. All data analyses were performed using Prism 7.0 and R (v.4.0.2, https://cran.r-project.org/). All statistical tests were two-sided and P-values < 0.05 were siginificant, Kaplan–Meier method was used for survival analysis.

Thirty-four previously untreated patients who received iFCR treatment between January 2019 to March 2021 were enrolled. The median age was 55 years old (interquartile range: 48–56). Regarding the biological characteristics, 61.8% (21/34) patients had unmutated IGHV and 32.4% (11/34) patients had CK. TP53 mutation was detected in 17.6% (6/34) patients, including five with del(17p) (Supplementary Table 1). One patient received 2 cycles of iFCR, 13 received 3 cycles, 10 received 4 cycles, 1 received 5 cycles and 9 received 6 cycles of iFCR and the treatment schemes were shown in Supplement Fig. 1. At the data cut-off (August 1st, 2022), 27 (27/34, 79.4%) patients completed 24 cycles. Among them, six (6/34, 17.6%) patients discontinued ibrutinib after 24 cycles due to sustainable CR and peripheral blood (PB) & BM-uMRD. Four (4/34, 11.8%) patients progressed during ibrutinib maintenance.

The best ORR was 100% and the best response rate of CR/CRi, PB-uMRD, BM-uMRD, and CR/CRi with BM-uMRD were 73.5% (25/34), 76.5% (26/34), 73.5% (25/34) and 61.8% (21/34), respectively (Fig. 1a). After the median follow-up of 33 (range 7–42) months, 64.7% (22/34), 52.9% (18/34), 50.0% (17/34), and 47.1% (16/34) of patients sustained CR/CRi, PB-uMRD, BM-uMRD, and CR/CRi with BM-uMRD. The 2-year PFS and OS rates were 96.8% and 100%, and the 3-year PFS and OS rates were 80.0% and 95.5% (Fig. 1b). Regarding IGHV mutation, no differences in the best response rate of CR/CRi, PB-uMRD, and BM-uMRD were found between the unmutated IGHV subgroup without TP53 aberration and mutated IGHV subgroup (86.7% vs. 84.6%) (Fig. 1a). In the subgroup analyses of response at the last follow-up, TP53 aberration was the only adverse factor for patients achieving sustainable CR/CRi with BM-uMRD (*p* = 0.011) and CR/CRi alone (*p* < 0.001) and showed the same tendency for patients failing to achieve sustainable BM-uMRD (*p* = 0.175) during follow-up (Fig. 1c and Supplementary Fig. 2).

**Fig. 1:**
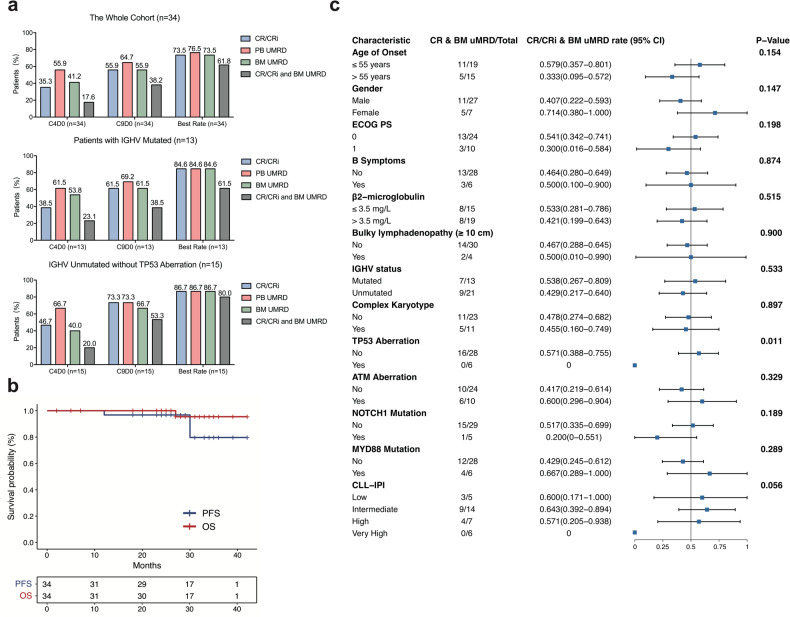
Response assessment for all 34 patients. **a** Response and MRD were analyzed in all patients and separately for those with mutated IGHV, and those with unmutated IGHV without TP53 aberration. **b** Progression-free survival and overall survival for all 34 patients. **c** Subgroup analysis of CR/CRi with BM-uMRD at last follow-up.

For 23 patients who received 3–4 cycles of iFCR, CR/CRi rate and BM-uMRD rate increased from 47.8% (11/23) and 60.9% (14/23), respectively after 3 cycles to 73.9% (17/23) and 60.9% (14/23), respectively after 6 cycles and sustained at 73.9% (17/23) and 56.5% (13/23), respectively till last follow-up (Fig. 2a). Three patients who were further confirmed BM-MRD6 by NGS-MRD assay after 3 cycles of iFCR achieved sustainable CR/CRi with BM-MRD6 during follow-up (Supplementary Fig. 3). Nine patients completed 6 cycles of iFCR, and among them, five (55.6%) patients with BM-MRD3 or MRD2 at C4D0 achieved BM-uMRD after 3 additional cycles of iFCR while four (44.4%) patients with PR and BM-MRD > 1% failed to benefit from 3 additional cycles of iFCR (Fig. 2a). Baseline differences between patients who received 3–4 cycles of iFCR and patients who received 6 cycles were shown in Supplementary Table 2. Four patients underwent progression and the preexisting clones such as TP53, NOTCH1, and EGR2 mutation exhibited linear clonal evolution. Two patients evolved ibrutinib-resistant clones as BTK C481S and mutation in BTK immediate downstream partner PLCG2 and such clones were predominantly selected by ibrutinib treatment pressure (Fig. 2b).

**Fig. 2:**
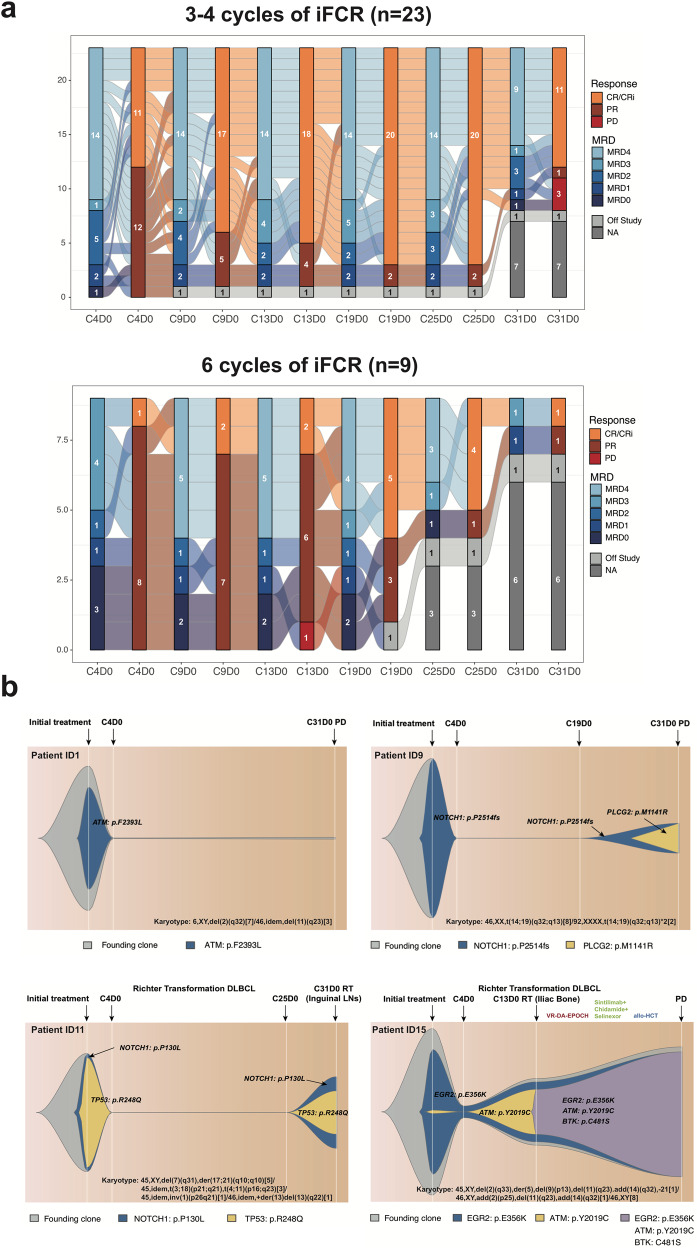
Subgroup analysis of patients who underwent remission and progression during follow-up. **a** Sankey diagram showing the dynamic change of BM-MRD levels and corresponding response assessment in patients who received three or four cycles of iFCR (*n* = 23) and in those who received six cycles of iFCR (*n* = 9); **b** Possible clonal evolution models of patients who progressed during treatment.

All patients experienced at least one episode of AE. The most common any-grade AEs were neutropenia (25/34, 73.5%), thrombocytopenia (24/34, 70.6%), and nausea (21/34, 61.8%). Hematological and non-hematological AEs were listed in Supplementary Table 3. 35.3% (12/34) of patients experienced at least one FC dose reduction and the most frequent reason was myelosuppression (*n* = 11). 47.1% of patients (16/34) experienced at least once ibrutinib dose-hold with a median duration of 7 days. 11.8% of patients (4/34) experienced at least twice ibrutinib dose-holds. 32.4% of patients (11/34) experienced at least one ibrutinib dose-reduction. Among totally 22 ibrutinib dose-hold events and 19 ibrutinib dose-reduction events, 36.4% (8/22) and 36.8% (7/19) occurred during iFCR treatment while 63.6% (14/22) and 63.2% (12/19) occurred during ibrutinib maintenance, respectively. No t-MDS/AML was found in our cohort. The incidences of grade 3–4 neutropenia and thrombocytopenia during ibrutinib maintenance in patients with 3–4 cycles of iFCR were lower than in patients with 6 cycles of iFCR. The patients who underwent 3–4 cycles of iFCR showed earlier recovery of serum IgG/IgA concentration and absolute CD4/CD8 T lymphocytes counts after six cycles (Supplementary Fig. 4).

In this cohort, we prospectively analyzed iFCR as the initial treatment in CLL/SLL, irrespective of the IGHV or TP53 status. Our study demonstrated that the iFCR regimen was highly effective in treatment-naïve CLL/SLL and could produce long-term remission and BM-uMRD in patients without TP53 aberrations. Our study emphasized patients with higher levels of BM-MRD ( ≥ 1%) after 3 cycles of iFCR could not achieve BM-uMRD even with more cycles of CIT.

In our study, the efficacy in patients without TP53 aberrations was not affected by IGHV mutational status and comparable to iFCR trial, which was the first one exploring 6 cycles of iFCR and two-year ibrutinib maintenance in CLL patients [3, 8]. The relatively higher BM-uMRD rate in the iFCG cohort as compared to our cohort could partly be attributed to excluding patients with TP53 aberrations and the additional benefit of obinutuzumab in eradicating MRD in BM [4, 9]. In the fixed-duration cohort of CAPTIVATE trial, patients with del(17p)/mutated TP53 could attain comparable CR/CRi rate (55% vs 56%) and relatively lower BM-uMRD rate (41% vs 62%) compared with patients without del(17p)/mutated TP53 [10, 11]. Although patients without TP53 aberrations in our cohort showed comparable CR/CRi rate, BM-uMRD rate, and CR/CRi with BM-uMRD rate to patients receiving ibrutinib plus venetoclax combination for 12 months in CAPTIVATE trial, patients with TP53 aberrations failed to achieve sustainable CR/CRi with BM-uMRD during long-term follow-up.

Subgroup analysis revealed that for most patients who achieved BM-uMRD after 3–4 cycles of iFCR, ceasing further FC regimen led to sustained or further remissions, while additional 3 cycles of FC regimen failed to benefit patients with BM-MRD ≥ 1%. Consistent with the results in iFCG trial [9], patients who were confirmed BM-MRD6 by NGS-MRD assay after 3 cycles of iFCR sustained BM-MRD6 during follow-up, suggesting the long-term clinical significance of achieving deep MRD remission in the early phase. Clonal evolution also indicated that CLL driving clones including TP53, NOTCH1, and EGR2 mutation and ibrutinib-resistant subclones such as BTK C481S and PLCG2 mutation led to progression under such combination regimen.

The present study had several limitations. It was a single-center real-world study with a limited number of patients. Lack of randomization to patients who received 3–4 to 6 cycles of iFCR and a relatively short follow-up of 33 months. Despite that, the present study demonstrated the durable effectiveness and favorable safety profile of the iFCR combination regimen in a real-world young fit Asian population of CLL/SLL without TP53 aberrations, irrespective of the IGHV mutational status.

### Supplementary information

Supplementary materials

## Data Availability

Original datasets will be made available by the authors upon reasonable request to the corresponding author (Huayuan Zhu, the First Affiliated Hospital of Nanjing Medical University, Jiangsu Province Hospital, Nanjing, Jiangsu, China; huayuan.zhu@hotmail.com).
