# Supplementary material for: Ibrutinib plus fludarabine, cyclophosphamide and rituximab (iFCR) as initial treatment in chronic lymphocytic leukemia/ small lymphocytic leukemia with or without TP53 aberrations: a prospective real-world study in Chinese cohort
Source: Blood Cancer J. 2023 Aug 9;13(1):121. doi: 10.1038/s41408-023-00890-y (PMC10412547; doi:10.1038/s41408-023-00890-y)
Supplement: Supplementary file 1 — Supplementary materials [file 41408_2023_890_MOESM1_ESM.docx]

**Supplementary Table 1** Baseline characteristics of the 34 patients at study entry.

|  | Number (%) or median (IQR) |
| --- | --- |
| Age, years | 55.0（48.0-57.0） |
| Gender, male | 27/34 (79.4%) |
| ECOG-performance score |  |
| 0 | 24/34 (70.6%) |
| 1  iwCLL classification  CLL  SLL | 10/34 (29.4%)  32/34 (94.1%)  2/34 (5.9%) |
| Rai stage (n=32) |  |
| 0 | 1/32 (3.1%) |
| I-II | 11/32 (34.4%) |
| III-IV | 20/32 (62.5%) |
| Binet stage (n=32) |  |
| A | 2/32 (6.2%) |
| B | 11/32 (34.4%) |
| C | 19/32 (59.4%) |
| B symptoms | 6/33 (18.2%) |
| Baseline labs |  |
| Absolute neutrocyte count (×10^9^/L) | 4.3 (2.4-7.1) |
| Absolute lymphocyte count (×10^9^/L) | 53.5 (17.0-126.7) |
| Hemoglobin (g/dL) | 115.5 (92.0-123.0) |
| Platelets (×10^9^/L) | 117.0 (87.0-138.0) |
| β2-microglobulin (mg/L) | 3.7 (3.0-5.0) |
| Bulky lymphadenopathy |  |
| 5-10 cm | 6/34 (17.6%) |
| ≥ 10 cm | 4/34 (11.8%) |
| IGHV status  Unmutated  Mutated | 21/34 (61.8%)  13/34 (38.2%) |
| Baseline FISH |  |
| Del(17p) | 5/34 (14.7%) |
| Del(11q) | 7/34 (20.6%) |
| Del(13q) | 16/33 (48.5%) |
| Trisomy 12 | 4/30 (3.3%) |
| Complex karyotype | 11/34 (32.4%) |
| Baseline gene mutation |  |
| TP53 | 6/34 (17.6%) |
| NOTCH1  SF3B1  ATM  MYD88 | 5/34 (14.7%)  2/34 (5.9%)  8/34 (23.5%)  6/34 (17.6%) |
| Del(17p) & TP53 mutation | 5/34 (14.7%) |
| CLL-IPI (n=32) |  |
| Low | 5/32 (15.6%) |
| Intermediate | 14/32 (43.8%) |
| High | 7/32 (21.9%) |
| Very High | 6/32 (18.7%) |

IQR: interquartile range; ECOG: Eastern Cooperative Oncology Group; iwCLL, international workshop on chronic lymphocytic leukemia; CLL, chronic lymphocytic leukemia; SLL, small lymphocytic lymphoma; IGHV: immunoglobulin heavy-chain variable region gene; FISH: Fluorescence in situ hybridization; CLL-IPI: international prognostic index for chronic lymphocytic leukemia.

**Supplementary Table 2** Baseline Characteristics of patients who received 3-4 versus 6 cycles.

|  | 3-4 cycles of iFCR (n=23) | 6 cycles of iFCR (n=9) | *p* value | |
| --- | --- | --- | --- | --- |
| Age, years | 55.0（51.0-56.0） | 52.0 (42.0-57.0) | 0.216 |  |
| Gender, male | 17/23 (73.9%) | 8/9 (88.9%) | 0.357 |  |
| ECOG-performance score |  |  | 0.491 |  |
| 0 | 15/23 (65.2%) | 7/9 (77.8%) |  |  |
| 1 | 8/23 (34.8%) | 2/9 (22.2%) |  |  |
| Rai stage |  |  | 1.000 |  |
| 0 | 1/21 (4.8%) | 0/9 (0.0%) |  |  |
| I-II | 8/21 (38.1%) | 3/9 (33.3%) |  |  |
| III-IV | 12/21 (57.1%) | 6/9 (66.7%) |  |  |
| Binet stage |  |  | 0.572 |  |
| A | 1/21 (4.8%) | 1/9 (11.1%) |  |  |
| B | 7/21 (33.3%) | 4/9 (44.4%) |  |  |
| C | 13/21 (61.9%) | 4/9 (44.4%) |  |  |
| B symptoms | 5/23 (21.7%) | 0/9 (0.0%) | 0.128 |  |
| β2-microglobulin (mg/L) | 3.4 (2.9-4.9) | 4.6 (3.6-5.5) | 0.098 |  |
| Bulky lymphadenopathy |  |  | 0.074 |  |
| 5-10 cm | 3/23 (13.0%) | 3/9 (33.3%) |  |  |
| ≥ 10 cm | 1/23 (4.3%) | 2/9 (22.2%) |  |  |
| IGHV status  Unmutated  Mutated | 13/23 (56.5%)  10/23 (43.5%) | 7/9 (77.8%)  2/9 (22.2%) | 0.264 |  |
| Baseline FISH |  |  |  |  |
| Del(17p) | 2/23 (8.7%) | 2/9 (22.2%) | 0.298 |  |
| Del(11q) | 4/23 (17.4%) | 2/9 (22.2%) | 0.753 |  |
| Del(13q) | 10/23 (43.5%) | 5/9 (55.6%) | 0.538 |  |
| Trisomy 12 | 4/20 (20.0%) | 0/9 (0.0%) | 0.148 |  |
| Complex karyotype | 6/23 (26.1%) | 5/9 (55.6%) | 0.115 |  |
| Baseline gene mutation |  |  |  |  |
| TP53 | 3/23 (13.0%) | 2/9 (22.2%) | 0.520 |  |
| NOTCH1  SF3B1  ATM  MYD88 | 5/23 (21.7%)  1/23 (4.3%)  5/23 (21.7%)  4/23 (17.4%) | 0/9 (0.0%)  0/9 (0.0%)  3/9 (33.3%)  1/9 (11.1%) | 0.128  0.525  0.496  0.660 |  |
| Del(17p) & TP53 mutation | 2/23 (8.7%) | 2/9 (22.2%) | 0.298 |  |
| CLL-IPI |  |  | 0.177 |  |
| Low | 5/21 (23.8%) | 0/9 (0.0%) |  |  |
| Intermediate | 10/21 (47.6%) | 3/9 (33.3%) |  |  |
| High | 3/21 (14.3%) | 4/9 (44.4%) |  |  |
| Very High | 3/21 (14.3%) | 2/9 (22.2%) |  |  |

IQR: interquartile range; ECOG: Eastern Cooperative Oncology Group; IGHV: immunoglobulin heavy-chain variable region gene; FISH: Fluorescence in situ hybridization; CLL-IPI: international prognostic index for chronic lymphocytic leukemia.

**Supplementary Table 3** Adverse events in the 34 patients.

| Events | n (%) |  |
| --- | --- | --- |
|  | Grade 1-2 | Grade 3-4 |
| Hematological |  |  |
| Anemia | 15 (44.1%) | 5 (14.7%) |
| \| Febrile neutropenia \| \| --- \| | 0 (0.0%) | 8 (23.5%) |
| Neutropenia | 2 (5.9%) | 23 (67.6%) |
| Thrombocytopenia | 12 (35.3%) | 12 (35.3%) |
| Non-hematological |  |  |
| Fatigue | 16 (47.1%) | 0 (0.0%) |
| Nausea | 19 (55.9%) | 2 (5.9%) |
| Vomiting | 13 (38.3%) | 2 (5.9%) |
| Arthralgia | 9 (26.5%) | 4 (11.8%) |
| Skin rashes | 13 (38.2%) | 2 (5.9%) |
| Purpura | 3 (8.8%) | 1 (2.9%) |
| Diarrhea | 10 (29.4%) | 2 (14.7%) |
| Lung infection | 1 (2.9%) | 5 (11.8%) |
| Urinary tract infection | 0 (0.0%) | 1 (2.9%) |
| Upper respiratory tract infection | 2 (5.9%) | 0 (0.0%) |
| Skin infection | 2 (5.9%) | 1 (2.9%) |
| Herpes zoster | 0 (0.0%) | 2 (5.9%) |
| Other infection | 2 (5.9%) | 0 (0.0%) |
| Hematuria | 12 (35.3%) | 0 (0.0%) |
| Atrial fibrillation | 0 (0.0%) | 2 (5.9%) |
| Headache | 2 (5.9%) | 0 (0.0%) |
| Back pain | 1 (2.9%) | 0 (0.0%) |
| Pain | 2 (5.9%) | 0 (0.0%) |
| Oral bleeding | 3 (8.8%) | 1 (2.9%) |

**Supplementary Figure 1** Flow chart of the 34 treated patients.


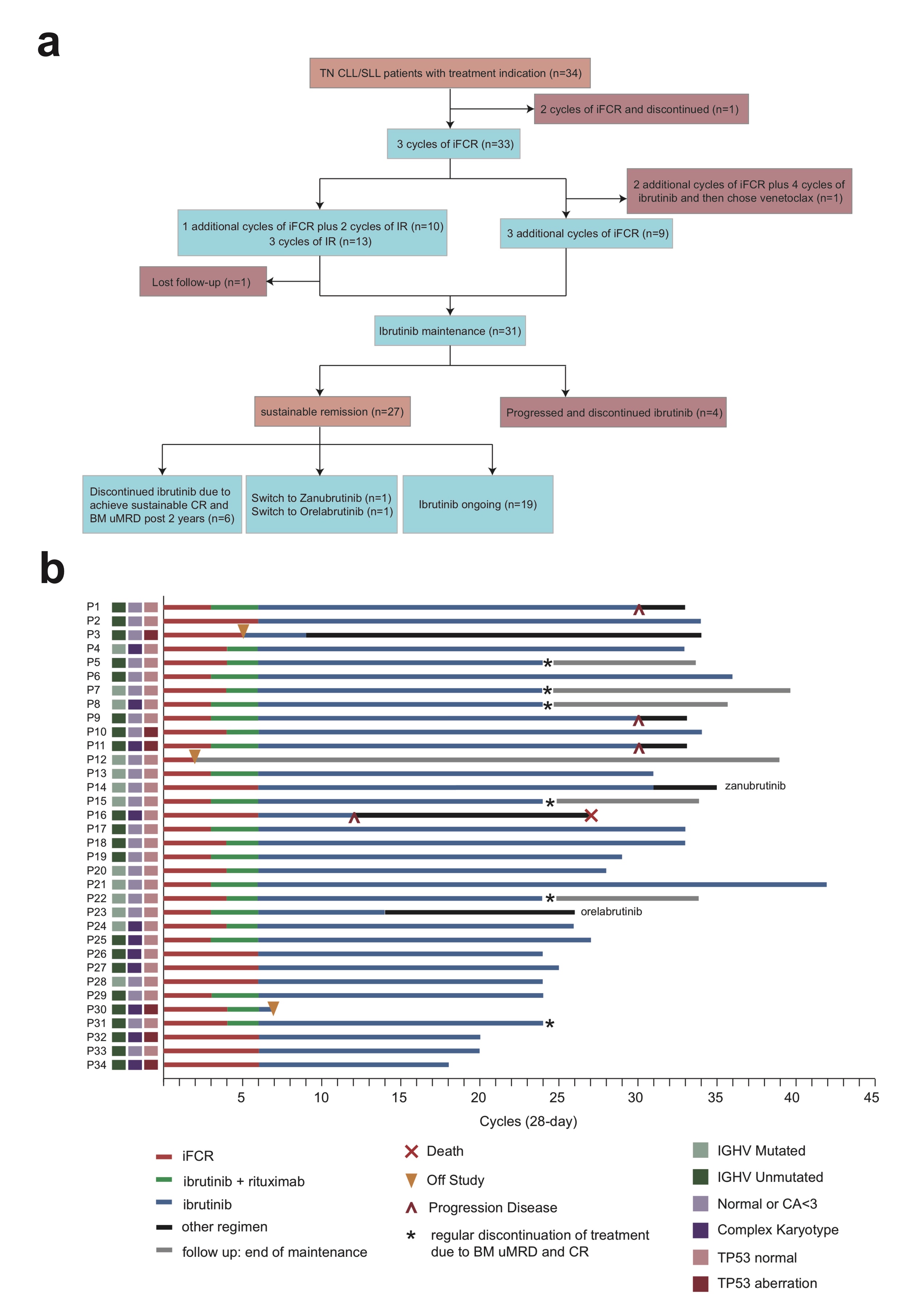


(a) Of the 34 treated patients, one discontinued treatment and underwent splenectomy after two cycles of iFCR due to splenomegaly. Thirteen patients received three cycles of iFCR and additional three cycles of IR. Ten patients received four cycles of iFCR and additional two cycles of IR. One patient received five cycles of iFCR plus four cycles of ibrutinib monotherapy and started to combine venetoclax with ibrutinib. Nine patients received six cycles of iFCR. Thirty-one patients then turned to ibrutinib maintenance. At the data cut-off (August 1^st^, 2022), 27 patients had sustained remission while four patients showed disease progression (including two patients who underwent Richter Transformation at cycles 12 and 30, respectively, and another two patients progressed at 30 cycles). Among 27 patients, six discontinued ibrutinib after 24 cycles due to CR and BM uMRD, while 21 patients sustained monotherapy treatment (one patient switched to zanubrutinib due to recurring bleeding events at cycle 25, one switched to orelabrutinib due to grade 3 hypofibrinogenemia at cycle 12, while 19 patients maintained ibrutinib treatment). (b) Swimmer’s plot of the 34 patients. Twenty-one patients had IGHV unmutated status (dark green dots), 11 had complex karyotype (dark purple dots), and six had TP53 aberration [five patients had TP53 mutation concurrent with del(17p), and one only harbored TP53 mutation, dark red dots]. Six patients were marked with black asterisks for regular treatment discontinuation due to CR and BM uMRD. Four patients were marked with red sharped angle as progression disease during follow-up (P1 and P9 progressed at cycle 30, P11 and P16 underwent Richter transformation at cycle 30 and 12, respectively, and P16 ceased at cycle 27). TN: Treatment-naïve; CLL/SLL: Chronic Lymphocytic Leukemia/Small Lymphocytic Lymphoma; iFCR: ibrutinib plus fludarabine, cyclophosphamide, and rituximab; IR: ibrutinib plus rituximab; CR: complete remission; BM: bone marrow; uMRD: undetectable minimal residue disease with 10^-4^ sensitivity; P: patient; IGHV: immunoglobulin heavy-chain variable region gene; CA: chromosomal aberrations.

**Supplementary Figure 2** Effect of IGHV mutational status, chromosomal abnormalities and TP53 aberrant status on the effectiveness at each time point.


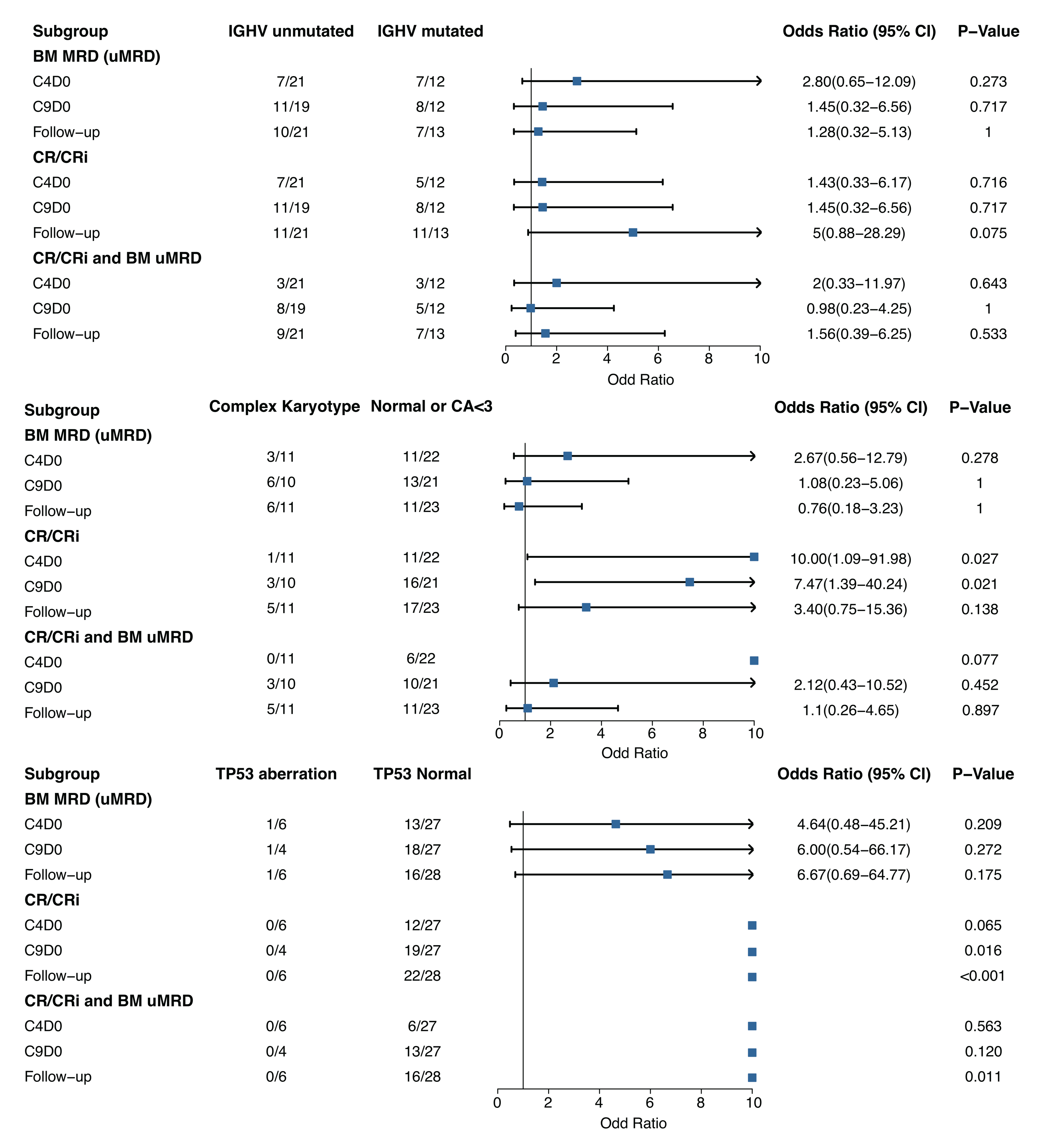


Subgroup analysis of the association of IGHV mutational status, chromosomal abnormalities, and TP53 status (mutation and deletion) with patients who achieved BM uMRD, CR/CRi and CR/CRi with BM uMRD at C4D00, at C9D0, and during long-term follow-up.

**Supplementary Figure 3 NGS** **MRD response at each time point.**

**
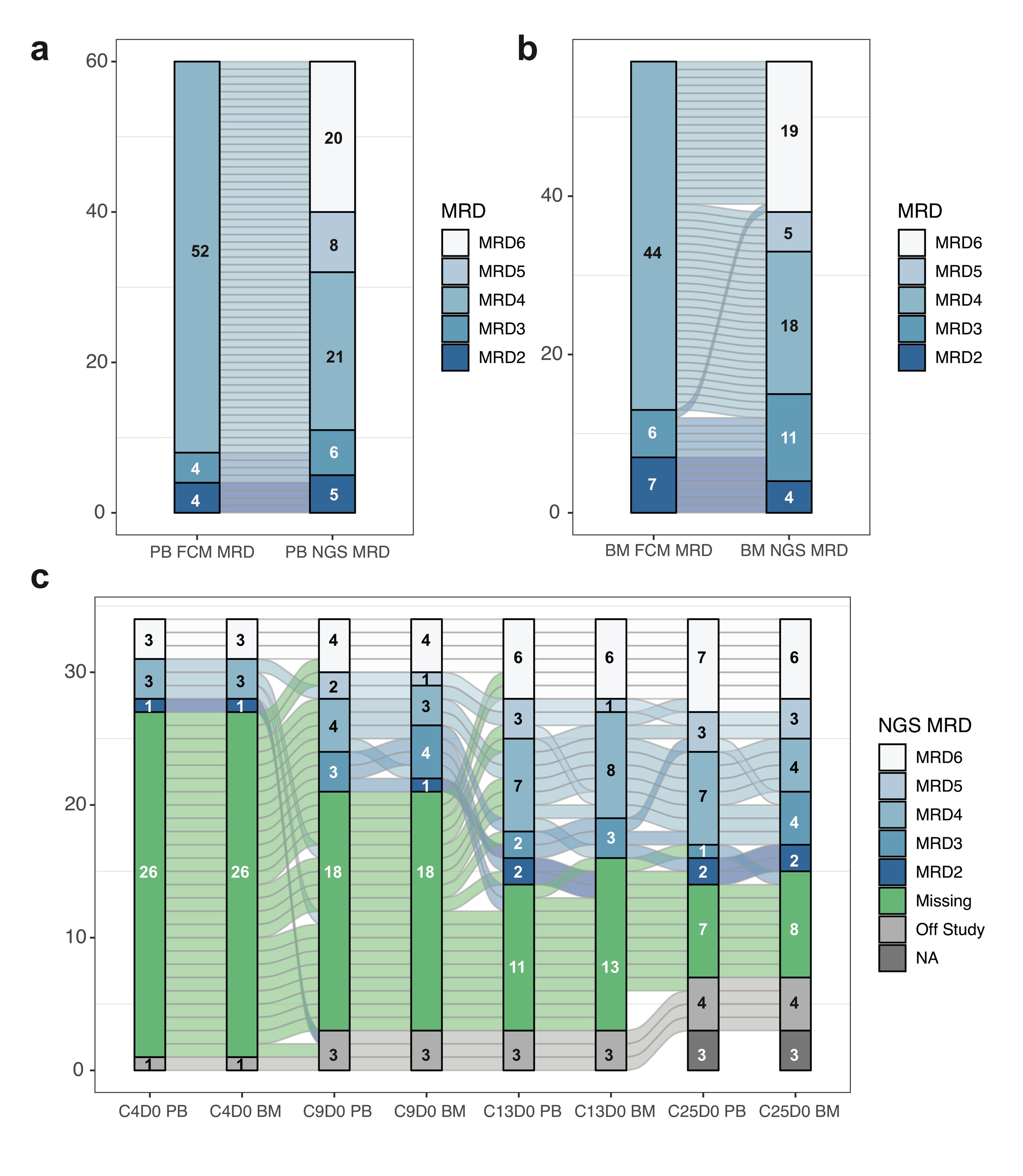
**

(a) PB MRD response by NGS assay versus FCM assay. (b) BM MRD response by NGS assay versus FCM assay. (c) Sankey diagram showing the dynamic change of corresponding PB and BM MRD change by NGS assay. Assessments were done after 3 cycles of iFCR (C4D0), 2 months after completion of six cycles of combined therapy (C9D0), after 12 cycles (C13D0), after 18 cycles (C19D0) and after 24 cycles (C25D0).

**Supplementary Figure 4** Hematological adverse effects and immune recovery status of patients who received three or four cycles of iFCR and six cycles of iFCR.


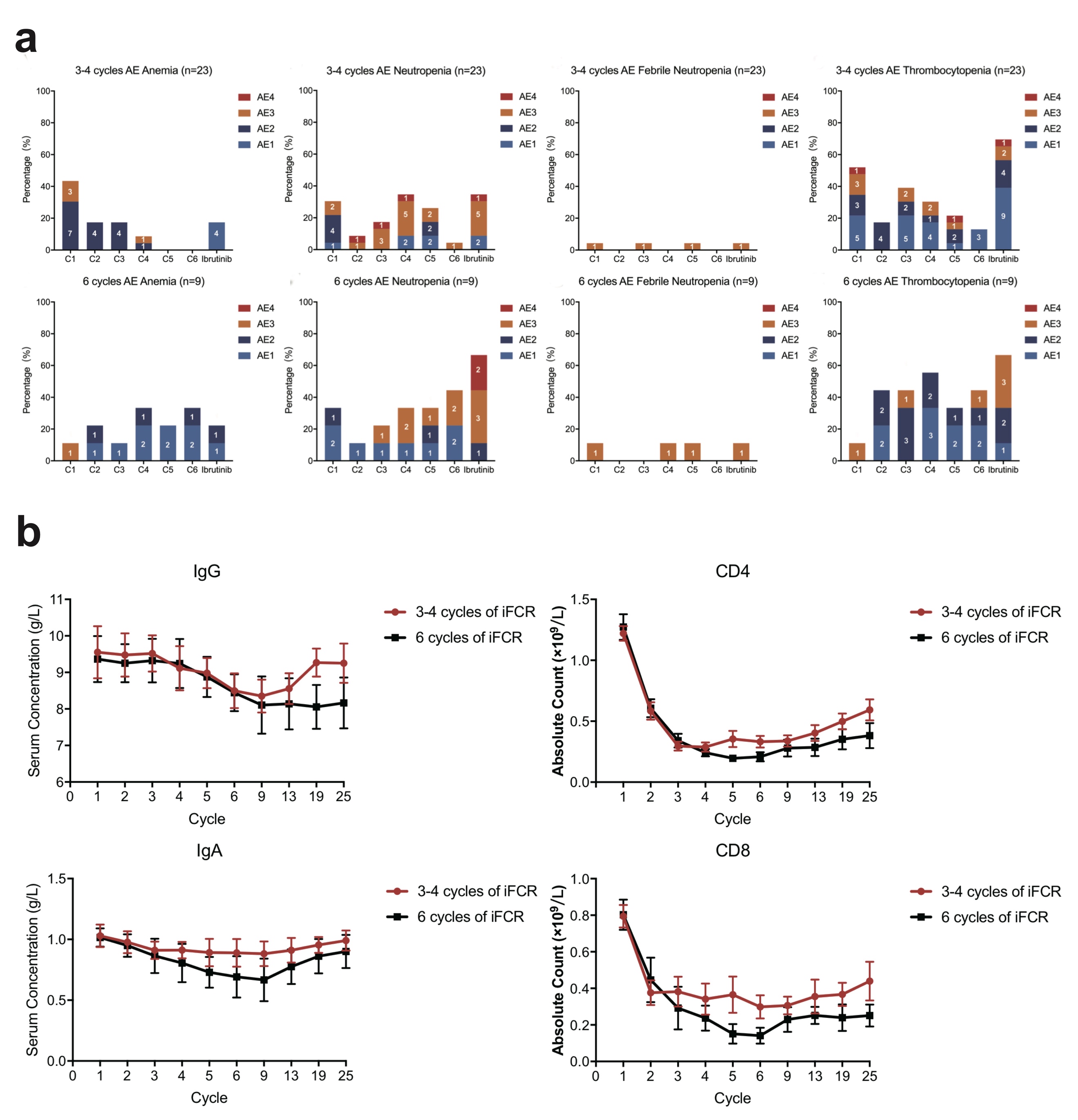


(a)Hematological adverse effects (AEs) of all grades occurred in each cycle and ibrutinib maintenance among 23 patients who received three or four cycles of iFCR and 9 patients who received six cycles of iFCR, including anemia, neutropenia, febrile neutropenia, and thrombocytopenia; (b) Dynamic change of IgA, IgG serum concentration and absolute counts of CD4-positive and CD8-positive T cells in peripheral blood in each cycle during the treatment course.
